# Nurses’ Knowledge and Attitudes Regarding Pain Assessment and Management in Saudi Arabia

**DOI:** 10.3390/healthcare10030528

**Published:** 2022-03-14

**Authors:** Khaled M. AL-Sayaghi, Hammad A. Fadlalmola, Wael A. Aljohani, Ali M. Alenezi, Dalal T. Aljohani, Thana A. Aljohani, Sameer A. Alsaleh, Khalid A. Aljohani, Mohammed S. Aljohani, Naif S. Alzahrani, Ayman A. Alamri, Amraa H. Alhousah, Mumtaz F. Khan

**Affiliations:** 1 Department of Medical Surgical Nursing, College of Nursing, Taibah University, Al-Madinah Al-Munawarah 42353, Saudi Arabia; msejohani@taibahu.edu.sa (M.S.A.); nzahrani@taibahu.edu.sa (N.S.A.); 2 Nursing Division, Faculty of Medicine and Health Sciences, Sana’a University, Sana’a 1247, Yemen; 3 Department of Community Health Nursing, College of Nursing, Taibah University, Al-Madinah Al-Munawarah 42353, Saudi Arabia; hazzminno345@gmail.com (H.A.F.); kajohani@taibahu.edu.sa (K.A.A.); 4 Nursing Administration Department, King Fahad Hospital, Al-Madinah Al-Munawarah 42351, Saudi Arabia; waaljohani@moh.gov.sa; 5 Nursing Audit Department, King Fahad Hospital, Al-Madinah Al-Munawarah 42351, Saudi Arabia; al_enazi555@hotmail.com (A.M.A.); ayaalamri@moh.gov.sa (A.A.A.); 6 Nursing Education and Research Department, King Fahad Hospital, Al-Madinah Al-Munawarah 42351, Saudi Arabia; dalalta@moh.gov.sa (D.T.A.); thanaa@moh.gov.sa (T.A.A.); saalsaleh@moh.gov.sa (S.A.A.); aalhousah@moh.gov.sa (A.H.A.); mumtazm@moh.gov.sa (M.F.K.)

**Keywords:** nurses’ knowledge, nurses’ attitudes, pain assessment, pain management, Saudi Arabia

## Abstract

Inadequate pain management affects the patient outcome. Pain assessment and management are fundamental in nursing care, and nurses must be equipped with adequate knowledge and a positive attitude toward pain assessment and management. This study aims to evaluate nurses’ knowledge and attitudes regarding pain assessment and management at King Fahad Hospital, Al-Madinah, Kingdom of Saudi Arabia. A quantitative, cross-sectional survey, using a self-administered questionnaire, was conducted from January to February 2020 with 660 registered nurses working in the Emergency Department, critical care units, inpatient and outpatient departments at King Fahad Hospital in Al-Medinah, Kingdom of Saudi Arabia. The data were analyzed with descriptive and inferential statistics. Of the 660 nurses, 291 responded, resulting in a response rate of 44.09%. The participants’ scores ranged from 17.7% to 100%, with a mean score 45.29%. The majority of the participants (70.1%) had a poor level of knowledge and attitudes (score < 50%). Nurses working in the outpatient department scored significantly higher than the group working in the Emergency Department and inpatient wards. Deficient knowledge and negative attitudes were found and nurses continue to underassess and undertreat pain. Nursing school curricula and in-service continuous education must equip nurses with the required knowledge and attitudes to enable them to manage pain effectively.

## 1. Introduction

According to the American Pain Society (APS) and the International Association for the Study of Pain (IASP), pain is “an unpleasant sensory and emotional experience associated with actual or potential tissue damage” [1,2]. Because pain is a multifaceted and subjective phenomenon influenced by an individual’s culture, beliefs, previous pain experience, and ability to cope [2,3], it is defined as “pain is whatever the experiencing person says it is, existing whenever he says it does”. Pain cannot be verified, and self-report is the only reliable measure to assess the presence and intensity of pain [1,4].

Pain is one of the most prevalent symptoms associated with the health disorders that nurses manage daily. Although it is a protective mechanism that makes people seek healthcare services, pain is a major stressor that influences a person’s physiological, psychosocial, emotional, and financial status [5,6]. Unalleviated pain triggers physiological and psychosocial stress responses that affect every system in the patient’s body, resulting in harmful effects [7]. These effects include fear, anxiety, sleep disturbance, hopelessness, weak memory, decreased cognitive function, social isolation, and a lowered quality of life [5,7,8]. Undertreated pain delays the mobilization of the patient, delays wounds healing, depresses immunity, slows recovery, increases the risk of complications, increases hospital stay, and healthcare costs [3,4,5,7,9]. Mismanaged pain has numerous costs for the patients, families, healthcare professionals, healthcare services, and the community [9,10].

Pain is a global health concern and a universal human experience affecting all races, genders, ages, geographical locations, and socioeconomic classes [11,12]. Approximately 50–80% of hospitalized patients suffer pain [12,13,14]. Ethically, the patient should not be allowed to suffer if the pain can be alleviated [15]. Suitable and effective pain treatment is a fundamental human right under the International Human Rights Law, and an essential step in enhancing the patient’s quality of life [10,15,16]. In recent decades, pain has become an important area of research and pain management a top priority in healthcare [7,17]. Despite the substantial growth in the body of knowledge, the availability of guidelines, and advances in technologies and various pharmacologic, as well as non-pharmacologic pain management approaches, all kinds of pain continue to be underestimated and undertreated and hospitalized patients still experience pain unnecessarily [5,7,13,18].

The effective management of pain is an important and sensitive indicator of the quality of nursing and healthcare [14,15]. Although achieving satisfactory pain relief needs interdisciplinary team collaboration and is an ethical and moral responsibility of all teams [10,11,19], nurses play a vital role in alleviating the patient’s pain as experts in pain assessment, management, and patient education in all healthcare settings [4,8,10]. The accurate assessment and effective management of pain require nurses to understand the concepts and acquire current knowledge, an appropriate attitude, and effective skills related to the assessment and management of pain [10,20]. The nurse must use a systematic process with a reliable and valid scale for precise pain assessment, believe and approve the reported pain level by the patients themselves, provide support, use pharmacological and nonpharmacological methods of pain management, and evaluate the effectiveness of the management [8,15,19]. Unfortunately, studies with nurses reported a lack of knowledge, attitude, and skills necessary for effective pain management. Pain continues to be misinterpreted, poorly estimated, and undermanaged in various healthcare settings globally [3,6,14].

A large proportion of the nurses employed in the Kingdom of Saudi Arabia (K.S.A.) are expatriates with a different educational background, experiences, culture, religion, and languages. Nursing knowledge and attitudes towards pain assessment and management are not well established in K.S.A., and more studies are required related to this critical topic. Although few, most of studies conducted in K.S.A. to assess the nurses’ knowledge and attitudes about pain management had some limitations, including recruiting various healthcare workers with a small group of nurses or recruiting only nurses, but with a small sample size [21,22,23,24], or nurses from only one or two nursing specialties [15,22,25,26]. Such limitations reduce the generalizability of the findings, and call for further studies. The aim of the current study is to assess the knowledge and attitudes of nurses regarding pain assessment and management in King Fahad Hospital in Al-Madinah Al-Munawarah, K.S.A.

## 2. Materials and Methods

### 2.1. Study Design

A quantitative, cross-sectional, descriptive design using a self-administered survey to assess the level of knowledge and attitudes of nurses towards the assessment and management of pain.

### 2.2. Setting and Participants

The study was conducted at King Fahad Hospital in Al-Medinah, K.S.A. King Fahad Hospital is the largest tertiary referral governmental hospital in the Al-Madinah AlMunawarah Region with a capacity of 500 beds. This study used a convenience sampling (nonprobability sampling) method and all the registered nurses working in the emergency department (ED), critical care units, general inpatient wards, and the outpatient department (OPD) were invited to participate in the study. Nurses were included regardless of their gender, nationality, and educational level. Newly hired nurses (less than two months), nursing students, and nurses from the operation rooms, nursing administration, and nursing education departments were not invited to participate. The sample size was calculated using the Raosoft sample size calculator [27]. The sample size was calculated based on a power level of 0.95 and a margin error of 0.05. For the 800 nurses working in the previously mentioned departments, a sample size of 260 was sufficient. In total, 660 nurses agreed to participate and received questionnaires of which 291 were returned, with a response rate of 44.09%.

### 2.3. Data Collection Instrument

A self-administered questionnaire containing two parts was used for data collection. The first part gathered the sociodemographic information. The second part assessed the knowledge and attitudes of the nurses regarding the assessment and management of pain. The Knowledge and Attitude Survey Regarding Pain (KASRP) standardized questionnaire was used. The KASRP was developed by Ferrell and McCaffery in 1987 and revised several times, with the latest version revised in 2014 by the original authors [28]. According to Ferrell and McCaffery, the validity and reliability have been established; the test–retest reliability of the KASRP was 0.80 and the internal consistency 0.70 (alpha r). The KASRP questionnaire is available in the public domain and is extensively used in various languages and in different contexts globally.

The KASRP questionnaire contains 41 items, including 22 true/false, 15 multiple-choice questions, and 4 additional multiple-choice questions based on 2 case scenarios. According to the KASRP author recommendations, the items are not distinguished as measuring attitude or knowledge because of the overlapping in many items that measure both variables together (knowledge and attitude). The two scenarios are identical with the exception of the patient’s behavior. The patient in scenario A was smiling, talking, and joking with his visitors, and the patient in scenario B was lying quietly and grimaced as he turned in the bed. The nurses were asked to assess the level of pain. Nurses were then asked to manage the pain in both scenarios, based on their assessment.

### 2.4. Data Collection

The KASRP was distributed to the nursing personnel after receiving approval from the Institutional Review Board (IRB), January to February 2020. Every nurse was provided with an envelope containing an information document, a consent form, and the questionnaire. If interested in participation, the nurse was required to sign the consent, complete the questionnaire, and submit the sealed envelope to the researchers. The data were collected from all participants in a period of one month. To complete the data collection, three reminders were sent to the nursing staff.

### 2.5. Ethical Issues

The current study was approval by the Research Ethical Committee and Institutional Review Board (IRB) at King Fahad Hospital. Consent was also obtained from the nursing administration. Participation was voluntary and participant confidentiality and anonymity were ensured. No names, ID numbers, email, or other identification data were collected. Each questionnaire received a code to protect the confidentiality. The questionnaires were stored in a locked place and the data kept in the personal computer of the main researcher and secured with a password.

### 2.6. Data Analysis

Data were analyzed using the Statistical Package for Sciences (SPSS version 16 (IBM, Armonk, NY, USA)). Descriptive statistical analysis was performed for gender, age, nationality, qualification, years of experience, and place of work. For the KASRP tool, correct responses were scored as “one” and incorrect responses scored as “zero” (total score ranged from 0–41). The data were analyzed in terms of individualized items and the number of correct responses for each item. The total score is provided in percentage of correct answers through dividing the total number correct responses by the total number of KASRP items (X/41). The scores < 50% were classified as poor, 50% to <75% acceptable, and ≥75 as good knowledge and attitude [8,17]. Normality was checked and showed no major deviation and the linearity measures confirmed between dependent and independent variables. The differences between the scores based on the characteristics were measured using an independent *t*-test and the ANOVA test. A post hoc test was performed when the ANOVA test was significant. *p* < 0.05 was considered significant at the statistic scale.

## 3. Results

### 3.1. Demographic and Educational Characteristics

The KASRP questionnaire was distributed to 660 nurses at King Fahad Hospital in Medina, 291 responses were collected, with a response rate of 44.09%. About half of the sample were Saudi (51.2%) and the majority (81.1%) were female. Most were below 40 years (89.3%), and 68.7% had a Bachelor or Master’s degree in nursing. Less than half (39%) had ≤5 years of experience, 36.4% 6 to 10 years, and 24.7% ≥11 years of experience in the nursing profession. A third (36%) were from the critical care units, 29.2% from inpatient wards and 19.9% from the ED (Table 1).

### 3.2. Responses

The frequency and percentage of the sample who responded correctly are available in Table 2. Of the 41 questions, for 25 items more than 50% responded incorrectly, However, 18 of the 25 items were incorrect for more than 70% of the sample. The 25 incorrect items were primarily related to the level of knowledge and attitude about cancer-related pain, pain assessment, and pharmacological interventions. Only 6 items (6/41) were correctly answered by >75% of the sample, which mainly focused on opioids and physical dependence and substance abuse.

Regarding the intensity of the pain in the two case scenarios (one patient expressed behavioral manifestations of pain and another had no behavioral manifestations), the correct level of pain in both scenarios was eight. In the first case scenario (A) that involved a smiling, joking, and talking patient, 73% scored the intensity of pain incorrectly and 87% selected the wrong intervention to manage the pain. There was an improvement in the second scenario (B) involving a quiet and grimacing patient. However, less than half (44%) scored the pain intensity correctly and only 22% selected the correct intervention to manage the pain, compared to 26.8% and 13.4% in the first scenario (A) (Table 2).

The participants’ score ranged from 7 to 41 (17.7% to 100%), with a mean total score of 18.57/41 (45.29/100). The majority of the participants (70.1%) had a poor level of knowledge and attitude (scored <50%), 29.2% acceptable (scored from 50 to <75%), and only 0.7% a good level of knowledge and attitude (≥75%). Only one participant provided 100% correct responses.

### 3.3. Effect of Nurses’ Characteristics on Their KASRP Score

The effect of the nurses’ characteristics on their knowledge and attitude scores is illustrated in Table 3. The analysis of variance revealed that the effect of the clinical setting on the knowledge and attitude scores was significant (*p* = 0.002). The post hoc test indicated that the nurses working in the OPD had a higher level of knowledge and attitude (49.55%) than the group working in the ED and inpatient departments. All other variables, including nationality, age, gender, educational level, or total work experience were non-significant.

## 4. Discussion

Untreated pain is a global healthcare issue resulting in avoidable complications and unnecessary healthcare expenditure. Health equity and justice in the provision of healthcare necessitate that all patients must receive comfort measures and be free from pain and suffering. The current descriptive cross-sectional study is the first of its size conducted in Al-Medinah Al-Munawarah Region to evaluate the knowledge and attitudes of nurses about pain. The findings increase the concern of low level of knowledge and inappropriate attitudes about the pain experienced by patients. The sample had a mean score of 45.29% with the KASRP tool. The majority (70.1%) of the participants had a poor level of knowledge and attitude (scored <50%), with only 0.7% displaying a good level of knowledge and attitude (≥75%). Similar findings were reported by Al-Quliti and Alamri [21], with 87 nurses in Al-Madinah hospitals (mean 40.31% and none scored above 60%). Samarkandi [24] reported a mean of 46% for nurses in Riyadh. Eid et al. [29] reported a mean score of 42.5% for the nurses at a tertiary teaching hospital in Jeddah, and Alqahtani and Jones [25] reported a mean score of 45.1% for oncology nurses at five large hospitals in K.S.A. Similarly, Alotaibi et al. [26] reported a mean score of 45.2% for pediatric nurses.

The knowledge and attitude deficits in the current study are similar to the results of a Mexican study (40%) [3], China (Hong Kong) (47.72%) [30], Bahrain (47.9%) [31], Jordan (41.8–48.25%) [5,10,32], Vietnam (45.2%) [8], and Eritrea (49.5%) [9]. However, the knowledge and attitude deficit in present study is lower than other studies globally (20.4–38.2%) [6,33,34,35], and higher than in studies outside of the K.S.A. (54.9–74%) [12,14,30,36]. Possible reasons for this inconsistency might be a difference in sampling techniques, sample size, data collection tools and methods, study setting, category and the qualification of the respondents, in-service pain management education, and outcome rating [3,33]. The racial, religious, cultural, and professional diversity of the nursing staff in K.S.A. could be another explanation for the inconsistency [25].

The problem of pain under-assessment and under-treatment persists and the patient’s suffering due to pain continues. Poor knowledge and a negative attitude about the assessment and management of pain might be the result of a lack of attention given to pain related topics and the lack of sufficient time devoted to this topic in nursing school curricula. This results in the insufficient preparation of nurses during their undergraduate education, as well as the lack of in-service education programs related to pain [3,8,30]. Nursing schools and educators have to focus on teaching and training nursing students in this vital subject. Educational curricula should be revised to include a dedicated unit with sufficient hours to teach the physiopathology, assessment, and management of pain, including pharmacological and non-pharmacological methods [3,5,7]. There is a definite need to improve the performance of nurses about the assessment and management of pain in hospitals. Establishing permanent periodical in-service educational and evaluation programs on pain assessment and treatment are a necessity to alleviate patient suffering in hospitals.

This study reported that 60% (25/41) of the KASRP items were answered incorrectly by >50% of the sample. This is consistent with Issa et al. [15], who found that >60% of the items were answered erroneously by >50% of critical care nurses in a Tertiary Hospital in Riyadh (King Saud Medical City). Similarly, Kahsay and Pitkäjärvi [9] reported that 54.3% of the items were correct by <50% of the sample, and 6 by <30% of nurses. Admass et al. [33] found that 19 of the 41 items were correctly answered by <50% of Ethiopian oncology nurses.

The comparison of the responses of some items revealed a disagreement between the nurses’ knowledge and beliefs and their pain assessment and management practices. Although 57.73% of the nurses agreed that pain is subjective and the best evaluator of the pain intensity is the self-report of the patient, 72% agreed to use a placebo (an ethically unaccepted practice) as a useful method to assess for the presence of real pain and patient credibility, which is in line with the literature [15,32,33]. The case scenarios demonstrated additional inconsistency as only 26.8% correctly assessed the level of pain of the smiling and joking patient, and only 43.64% the level of pain of the grimacing and quiet patient. When there were no behavioral manifestations of the pain, the sample tended to ignore the patient’s verbalization of the pain score in favor of the objective clinical assessment, which is supported in the literature [3,10,29]. Nurses devalue the patient’s self-report, believing the patient is exaggerating or seeking attention, rather than reporting real pain [6,9,26]. Confirming this negative attitude, 82.5% relied on the vital signs (as objective clinical indicators) to assess the pain intensity and assumed that stable vital signs is an indicator of lack of pain. This erroneous belief is not limited to the current study, as reported in the literature [15,24,31]. Pain is a unique subjective experience, so the appropriate attitude of the nurses must be not to disregard the patient’s complaint of pain even if he is smiling, joking, or grimacing [3,15,37]. The result of this inappropriate attitude would be disregarding or an underestimation of the patient’s self-report of pain, which affects the pain management [21].

The literature states that nurses’ exaggerated fears about patient respiratory depression and addiction, and their inability to differentiate between tolerance and dependence are barriers for appropriate pain management [5,23,24]. As reported by Nguyen et al. [8] and Admass et al. [33], this study found that only 28 nurses (9.6%) knew that the risk of respiratory depression increased by <1% when the Morphine IV dose increased from 200 mg to 250 mg/hours in a cancer patient, and only 16.49% knew the manifestations of opioid physical dependence. Surprisingly, even when the patient was recognized to have severe pain (grimacing patient in the second case scenario), he did not receive the appropriate amount of opioid analgesics (only 22% of nurses selected the correct dose of opioid), which is comparable to the literature [8,33]. The majority (74%) of the nurses agreed to allow the patient to suffer as much as possible before providing opioid analgesia and 82.5% agreed to keep the patient suffering to search for the pain source, consistent with Samarkandi [24], Nguyen et al. [8], Lui et al. [30], and Al Qadire and Al Khalaileh [32]. Patient suffering is not ethically acceptable and nurses must know that there is no value in maintaining the suffering, while searching for the source and no benefit to encourage the patient to endure pain [15].

The literature reports that nurses have a poor knowledge in pain pharmacotherapy as supported by the present study [3,14,26]. Only 24% of the sample responded correctly to the question related to the recommended routes for opioid analgesia administration for persistent cancer-related pain, which is supported in the literature [8,15]. The majority (73%) agreed that substance abuse patients should not have opioid analgesia if they are experiencing pain, which is ethically and clinically unfair, also reported by Alkhatib et al. [10], Alqahtani and Jones [25], and Issa et al. [15]. It is the patient’s right to receive the correct treatment to alleviate his pain, regardless of his substance abuse history. Poor knowledge regarding pain pharmacological interventions could be a result of erroneous beliefs that pain medication is a function of physicians and nurses have no role to impact the physicians’ prescription [10,20]. Even though the prescription of an opioid is not a nursing responsibility, nurses are in charge of the optimal interval, timing, and dose to be given, based on the individual’s response [9,20]. The hospital should provide intensive courses for nurses in clinical pharmacology as well as the pain related content in nursing Bachelor’s degrees [3,24].

The current study found that the clinical setting/area had a significant effect on the knowledge and attitude of the sample towards pain. The nurses working in OPD had a higher level of knowledge and attitude than the group working in the ED and inpatient departments. This may possibly be due to the nurses in OPD having enough time to focus on the patients’ pain, while in the other departments and units, such as the ED or critical care unit, the focus and the priority is life threatening conditions, airway, hemodynamic variables, and primary disease processes [9,38]. This finding is inconsistent with the results of D’emeh et al. [5], who reported that the nurses in the surgical departments scored higher than the nurses in other departments and units.

The analysis indicated that the other variables of nationality, age, gender, educational level, or total work experience were non-significant in terms of the knowledge and attitude scores. Liyew et al. [12], Yaqoob and Nasaif [31], Al Qadire and Al Khalaileh [32], and Al-Quliti and Alamri [21] reported similarly that age, gender, nationality, marital status, qualification, clinical area, and work experience had no significant effects on the score of the nurses’ knowledge and attitudes towards pain. However, Umuhoza et al. [11] reported that gender, age, marital status, qualification, and working experience significantly affected the knowledge of nurses. Brant et al. [14] found that nursing experience, qualification, and in-service education affect the nurses’ score, and Alqahtani and Jones [25] that nationality significantly affected the nurses scores. Admass et al. [33], and Kahsay and Pitkäjärvi [9] reported that the qualification significantly affected the knowledge and attitude scores of the nurses.

### Limitations

Although the KASRP is a well-known valid and reliable tool and implemented by many researchers globally, the current study has some limitations. The study was limited to one referral hospital in Al-Madinah City, which could have more opportunities for continuous education and more qualified staff. The finding may not be generalizable to all healthcare facilities in K.S.A. Additional multi-center studies with a larger sample are recommended. Evaluating the knowledge and attitude does not always reflect practice, and an evaluation of the nurses’ practice with an observational checklist or documents review is recommended. Lastly, our study did not evaluate the total hours of the nursing school curricula dedicated to the topic of pain assessment and management. Collecting such data in any future study could support an improved understanding of the low level of knowledge and attitude of the nurses.

## 5. Conclusions

Nurses have significantly poor knowledge and inappropriate attitudes to the assessment and management of pain. Although all had poor scores, nurses in the OPD had a significantly higher knowledge and attitude scores than the nurses in the ED and inpatient wards. The other demographic variables were non-significant. Serious interventions in the hospitals are essential and nursing schools should provide nurses with adequate, updated, and accurate knowledge about the assessment and management of pain.

## Figures and Tables

**Table 1:** Demographic and Educational Characteristics of the sample.

Characteristic	Participants	
	*N*	%
**Total Sample**	291	100
**Nationality**		
Saudi	149	51.2
Non-Saudi	142	48.8
**Gender**		
Male	55	18.9
Female	236	81.1
**Age (years)**		
<30	124	42.6
30–39	136	46.7
≥40	31	10.6
**Educational status**		
Diploma Degree	91	31.3
University Degrees	200	68.7
**Total work experiences (years)**		
≤5	113	38.8
6–10	106	36.4
≥11	72	24.7
**Area of assignment**		
Emergency Department	58	19.9
Inpatient Wards	85	29.2
Outpatient Department (OPD)	44	15.1
Critical Care Units	104	35.7

**Table 2:** Correct responses for each item of the KASRP (*n* = 291).

Item No.	Item	Nurses Answered Correctly	
		No	%
**Pain Assessment**			
31	The most accurate judge of… (the patient)	168	57.73
32	Which of the following describes the… (patient should be individually assessed to determine cultural influence)	150	51.55
39A (40)	Patient B: Robert is 25 years old… (8)	138	44.0
12	Children less than 11 years old cannot… (F)	110	37.80
2	Because their nervous system is underdeveloped…. (F)	107	36.77
4	Patients may sleep in spite of severe pain… (T)	92	31.62
3	Patients who can be distracted from pain usually… (F)	84	28.87
38A (38)	Patient A: Andrew is 25 years old… (8)	79	27.1
1	Vital signs are always reliable indicators of… (F)	51	17.53
**Cancer-related Pain**			
25	Which of the following analgesic medications… (morphine)	189	64.95
30	Which of the following is useful for… (all of the above)	135	46.39
5	Aspirin and other nonsteroidal anti-inflammatory… (F)	93	31.96
23	The recommended route of administration… (oral)	71	24.40
28	A patient with persistent cancer pain… (less than 1%)	28	9.62
**Pharmacology**			
14	After an initial dose of opioid analgesic… (T)	243	83.51
34	The time to peak effect for morphine… (15 min)	230	79.04
21	The term “equianalgesia” means approximately… (T)	224	76.98
7	Combining analgesics that work by different… (T)	221	75.95
13	Patients’ spiritual beliefs may lead them to… (T)	213	73.20
24	The recommended route of administration… (intravenous)	212	72.85
19	Benzodiazepines are not effective pain… (F)	202	69.42
27	Analgesics for postoperative pain… (around the clock on a fixed schedule).	190	65.29
6	Respiratory depression rarely occurs in patients… (T)	179	61.51
35	The time to peak effect for morphine… (1–2 h)	164	56.36
10	Elderly patients cannot tolerate opioids… (F)	148	50.86
29	The most likely reason a patient with… (the patient is experiencing increased pain)	144	49.48
16	Vicodin (hydrocodone 5 mg þ acetaminophen)… (T)	135	46.39
26	A 30 mg dose of oral morphine is… (Morphine 10 mg IV)	134	46.05
18	Anticonvulsant drugs, such as gabapentin… (F)	94	32.30
37	Which statement is true regarding opioid… (obstructive sleep apnea is an important risk factor)	84	28.87
8	The usual duration of analgesia of 1–2 mg morphine… (F)	84	28.87
9	Opioids should not be used in patients… (F)	80	27.49
15	Giving patients sterile water by injection… (F)	81	27.84
11	Patients should be encouraged to endure… (F)	76	26.12
39B(41)	Your assessment, above (grimacing pt)….(administer morphine 3 mg IV now)	65	22.3
17	If the source of the patient’s pain is unknown… (F)	51	17.53
38B (39)	Your assessment, above (smiling pt)… (administer morphine 3 mg IV now)	39	13.4
**Substance Abuse and Physical Dependence**			
22	Sedation assessment is recommended during….(T)	249	85.57
20	Narcotic/opioid addiction is defined as a chronic… (T)	236	81.10
33	How likely is it that patient who… (5–15%)	84	28.87
36	Following abrupt discontinuation of an opioid… (sweating, yawning, diarrhea and agitation with patients when the opioid is abruptly discontinued)	48	16.49

**Table 3:** Nurses’ mean scores based on their characteristics.

Characteristic	Score Mean (±SD)	*p*-Value
**Total Mean score**	45.29 (±9.86)	
**Nationality**		0.082
Saudi	44.03 (±9.75)	
Non-Saudi	46.32 (±9.89)	
**Gender**		0.636
Male	44.83 (11.29)	
Female	45.40 (9.52)	
**Age (years)**		0.163
<30	44.21 (8.74)	
30–39	46.47 (10.51)	
≥40	44.45 (10.81)	
**Educational status**		0.543
Diploma Degree	44.71 (10.33)	
University Degrees	45.56 (9.65)	
**Total work experiences (years)**		0.440
≤5	44.51 (10.05)	
6–10	45.37 (8.53)	
≥11	46.40 (11.71)	
**Area of assignment**		0.002 *
Emergency Department	43.73 (9.71)	
Critical Care Units	46.15 (11.00)	
Inpatient Wards	43.10 (8.99)	
Outpatient Department (OPD)	49.55 (7.03)	

* Post hoc test reveals that OPD nurses had significant higher knowledge and attitude than nurse in the emergency department and inpatient departments.

## Data Availability

Data generated during the study are presented in this paper. Raw data are, however, available from the corresponding author upon reasonable request and with the permission of the ethical committee at king Fahad Hospital.
